# Supplementary material for: Noise characteristics of the Escherichia coli rotary motor
Source: BMC Syst Biol. 2011 Sep 27;5:151. doi: 10.1186/1752-0509-5-151 (PMC3224245; doi:10.1186/1752-0509-5-151)
Supplement: Additional file 1 — Supplementary information. Supplementary information in .pdf format, including the extended model for the chemotaxis pathway, additional mathematical derivations and validity checks. [file 1752-0509-5-151-S1.PDF]

Supplementary information: Noise characteristics of the *Escherichia coli* rotary motor

Diana Clausznitzer[1,2,3] and Robert G. Endres*[1,2]

[1]Division of Molecular Biosciences, Imperial College London, SW7 2AZ London, UK
[2]Centre for Integrative Systems Biology and Bioinformatics, Imperial College London, UK
[3]BioQuant, Universität Heidelberg, 69120 Heidelberg, Germany

Email: r.endres@imperial.ac.uk;

*Corresponding author

Contents

1 Linearisation of full pathway model **1**

2 Response functions **2**

3 Noise spectra **3**

4 Effect of localisation of CheR on noise power spectrum **5**

5 Effect of CheR gene expression on noise power spectrum **6**

6 Effect of correlations in ligand concentration on noise power spectrum **7**

7 Number of high-frequency filters **8**

8 Alternative Master-equation approach **9**

9 Langevin model for motor dynamics **10**

1 Linearisation of full pathway model

Similar to the presentation for the simplified model in the main text, we linearise Eq. 1, 2 and 13-17 in the main text describing the full signalling pathway and insert the Fourier transforms of the dynamical

variables to obtain the Fourier transformed response functions $\hat{\chi}_R(\omega)$ and noise spectra $S_R(\omega)$ for the signalling pathway. The linearised equations read

$$\frac{d(\delta A_c)}{dt} = -\sum_j \frac{\partial A}{\partial M}\left(\lambda_1 \delta A_j + \lambda_9 \delta N_{B_p}\right) + \frac{\partial A}{\partial c}\frac{d(\delta c_j)}{dt} + \frac{\partial A}{\partial M}\eta_{M_j}(t) + \eta_{A_j}(t) \tag{1}$$

$$\frac{d(\delta N_{A_p})}{dt} = \lambda_2 \delta A_c - \lambda_3 \delta N_{A_p} + \lambda_4 \delta N_{Y_p} + \lambda_{10}\delta N_{B_p} + \eta_{A_p}(t) + \eta_{A,Y_p}(t) + \eta_{A,B_p}(t) \tag{2}$$

$$\frac{d(\delta N_{Y_p})}{dt} = \lambda_5 \delta N_{A_p} - \lambda_6 \delta N_{Y_p} - \eta_{A,Y_p}(t) + \eta_{-Y_p}(t) \tag{3}$$

$$\frac{d(\delta N_{B_p})}{dt} = \lambda_{11} \delta N_{A_p} - \lambda_{12} \delta N_{B_p} - \eta_{A,B_p}(t) + \eta_{-B_p}(t) \tag{4}$$

$$\frac{d(\delta X)}{dt} = \lambda_7 \delta N_{Y_p} - \lambda_8 \delta X + \eta_X(t) \tag{5}$$

with rate constants of the linearised model given in in the following table:

λ_i	expression
λ_1	$\gamma_R + \gamma_B {B_P}^{*2}$
λ_2	$\left(\frac{k_A}{N_C N}\right)(N_{A,tot} - N^*_{A_p})$
λ_3	$A^*_c\left(\frac{k_A}{N_C N}\right) + \left(\frac{k_y}{V_{cell}}\right)(N_{Y,tot} - N^*_{Y_p}) + \left(\frac{k_B}{V_{cell}}\right)(N_{B,tot} - N^*_{B_p})$
λ_4	$\left(\frac{k_Y}{V_{cell}}\right) N^*_{A_p}$
λ_5	$\left(\frac{k_Y}{V_{cell}}\right)(N_{Y,tot} - N^*_{Y_p})$
λ_6	$\left(\frac{k_Y}{V_{cell}}\right) N^*_{A_p} + k_{-Y}$
λ_7	$\frac{1}{V_{cell}}\left((1-P^*)\frac{\partial k_+}{\partial Y_p} - P^*\frac{\partial k_-}{\partial Y_p}\right)$
λ_8	${k_+}^* + {k_-}^*$
λ_9	$\frac{2\gamma_B A^* B^*_p}{V_{cell}}$
λ_{10}	$\left(\frac{k_B}{V_{cell}}\right) N^*_{A_p}$
λ_{11}	$\left(\frac{k_B}{V_{cell}}\right)(N_{B,tot} - N^*_{B_p})$
λ_{12}	$\left(\frac{k_B}{V_{cell}}\right) N^*_{A_p} + k_{-B}.$

2 Response functions

The response functions can be calculated from the linearised Eq. 1-5 without noise after inserting the Fourier transforms of the dynamical variables. The Fourier transformed response functions of CheA-P, CheY-P and the motor are

$$\hat{\chi}_{A_c}(\omega) = \frac{-i\omega N_C \frac{\partial A}{\partial c} - \lambda_9 N_C \frac{\partial A}{\partial M}\hat{\chi}_{N_{B_p}}(\omega)}{\lambda_1 \frac{\partial A}{\partial M} - i\omega} \tag{6}$$

$$\hat{\chi}_{N_{A_p}}(\omega) = \left(-i\omega\lambda_2 N_C \frac{\partial A}{dc}(\lambda_6 - i\omega)(\lambda_{12} - i\omega)\right)\cdot$$
$$\left\{\left(\lambda_1 \frac{\partial A}{\partial M} - i\omega\right)\cdot\left[(\lambda_3 - i\omega)(\lambda_6 - i\omega)(\lambda_{12} - i\omega) - \lambda_{10}\lambda_{11}(\lambda_6 - i\omega) + \right.\right.$$

$$
-\lambda_4\lambda_5\left(\lambda_{12}-i\omega\right)] + \lambda_2\lambda_9\lambda_{11}N_C\frac{\partial A}{\partial M}\left(\lambda_6-i\omega\right)\Big\}^{-1} \quad (7)
$$

$$
\hat{\chi}_{N_{Y_p}}(\omega) = \frac{\lambda_5}{\lambda_6-i\omega}\hat{\chi}_{N_{A_p}}(\omega) \quad (8)
$$

$$
\hat{\chi}_{X}(\omega) = \frac{\lambda_7}{\lambda_8-i\omega}\hat{\chi}_{N_{Y_p}}(\omega) \quad (9)
$$

$$
\hat{\chi}_{N_{B_p}}(\omega) = \frac{\lambda_{11}}{\lambda_{12}-i\omega}\hat{\chi}_{N_{A_p}}. \quad (10)
$$

From these equations we observe that CheA-P, CheY-P and the motor are in a cascade. At each level of the cascade, a new filter proportional to $(\lambda_i - i\omega)^{-1}$ is introduced which simply multiplies the response function of the previous level of the cascade. The characteristic frequencies λ_i contain the forward and backward rates of the relevant processes.

3 Noise spectra

The noise spectra can be calculated from the linearised Eq. 1-5. After inserting the Fourier transforms of the dynamical variables, calculating the absolute squared value and averaging, we obtain the noise spectra for CheA-P, CheY-P and the motor shown in Fig. 4 in the main text

$$
\begin{aligned}
S_{A_c}(\omega) = & \Big(\left|(\lambda_3-i\omega)(\lambda_6-i\omega)(\lambda_{12}-i\omega)-\lambda_4\lambda_5(\lambda_{12}-i\omega)-\lambda_{10}\lambda_{11}(\lambda_6-i\omega)\right|^2 \cdot \\
& N_C\left[\omega^2\left(\frac{\partial A}{\partial c}\right)^2 S_c(\omega)+\omega^2\left(S_a(\omega)+Q_M\right)\right]+ \\
& +\left|-\lambda_9 N_C\frac{\partial A}{\partial M}\left[(\lambda_3-i\omega)(\lambda_6-i\omega)-\lambda_4\lambda_5\right]\right|^2 Q_{-B_p}+ \\
& +\left|\lambda_9\lambda_{11}N_C\frac{\partial A}{\partial M}(\lambda_6-i\omega)\right|^2 Q_{A_p}+\left|\lambda_4\lambda_9\lambda_{11}N_C\frac{\partial A}{\partial M}\right|^2 Q_{-Y_p}+ \\
& +\left|\lambda_9 N_C\frac{\partial A}{\partial M}\left[\lambda_4\lambda_5+\lambda_{11}(\lambda_6-i\omega)-(\lambda_3-i\omega)(\lambda_6-i\omega)\right]\right|^2 Q_{A,B_p}+ \\
& +\left|\lambda_9\lambda_{11}N_C\frac{\partial A}{\partial M}\left[(\lambda_6-i\omega)-\lambda_4\right]\right|^2 Q_{A,Y_p}\Big)\cdot \\
& \Big|\left(\lambda_1\frac{\partial A}{\partial M}-i\omega\right)\left[(\lambda_3-i\omega)(\lambda_6-i\omega)(\lambda_{12}-i\omega)-\lambda_{10}\lambda_{11}(\lambda_6-i\omega)+\right. \\
& \left.-\lambda_4\lambda_5(\lambda_{12}-i\omega)\right]+\lambda_2\lambda_9\lambda_{11}N_C\frac{\partial A}{\partial M}(\lambda_6-i\omega)\Big|^{-2} \quad (11)
\end{aligned}
$$

$$
\begin{aligned}
S_{N_{A_p}}(\omega) = & \Big(N_C\lambda_2^2\omega^2\left(\frac{\partial A}{dc}\right)^2\left|(\lambda_6-i\omega)\left(\lambda_{12}-i\omega\right)\right|^2 S_c(\omega)+ \\
& +\left|\lambda_2(\lambda_6-i\omega)(\lambda_{12}-i\omega)\right|^2 N_C\left(\omega^2 S_a(\omega)+Q_M\right)+
\end{aligned}
$$

$$+\left|\left(\lambda_1\frac{\partial A}{\partial M}-i\omega\right)(\lambda_6-i\omega)(\lambda_{12}-i\omega)\right|^2 Q_{A_p}+$$
$$+\left|(\lambda_6-i\omega)\left(-\lambda_2\lambda_9 N_C\frac{\partial A}{\partial M}+\lambda_{10}\left(\lambda_1\frac{\partial A}{\partial M}-i\omega\right)\right)\right|^2 Q_{-B_p}+$$
$$+\left|\lambda_4\left(\lambda_1\frac{\partial A}{\partial M}-i\omega\right)(\lambda_{12}-i\omega)\right|^2 Q_{-Y_p}+$$
$$+\left|(\lambda_6-i\omega)(\lambda_{12}-i\omega)(\lambda_1\frac{\partial A}{\partial M}-i\omega)+(\lambda_6-i\omega)(\lambda_2\lambda_9 N_C\frac{\partial A}{\partial M}+\right.$$
$$\left.-\lambda_{10}\left(\lambda_1\frac{\partial A}{\partial M}-i\omega\right))\right|^2 Q_{A,B_p}+$$
$$\left.+\left|\left(\lambda_1\frac{\partial A}{\partial M}-i\omega\right)(\lambda_6-i\omega)(\lambda_{12}-i\omega)-\lambda_4\left(\lambda_1\frac{\partial A}{\partial M}-i\omega\right)(\lambda_{12}-i\omega)\right|^2 Q_{A,Y_p}\right)\cdot$$
$$\left|\left(\lambda_1\frac{\partial A}{\partial M}-i\omega\right)\cdot\left[(\lambda_3-i\omega)(\lambda_6-i\omega)(\lambda_{12}-i\omega)-\lambda_{10}\lambda_{11}(\lambda_6-i\omega)+\right.\right.$$
$$\left.\left.-\lambda_4\lambda_5(\lambda_{12}-i\omega)\right]+\lambda_2\lambda_9\lambda_{11}N_C\frac{\partial A}{\partial M}(\lambda_6-i\omega)\right|^{-2} \tag{12}$$

$$S_{N_{Y_p}}(\omega) = \frac{\lambda_5^2(\omega)}{|\lambda_6-i\omega|^2}\cdot\left(N_C\lambda_2^2\omega^2\left(\frac{\partial A}{dc}\right)^2|(\lambda_6-i\omega)(\lambda_{12}-i\omega)|^2 S_c(\omega)+\right.$$
$$+|\lambda_2(\lambda_6-i\omega)(\lambda_{12}-i\omega)|^2 N_C\left(\omega^2 S_a(\omega)+Q_M\right)+$$
$$+\left|\left(\lambda_1\frac{\partial A}{\partial M}-i\omega\right)(\lambda_6-i\omega)(\lambda_{12}-i\omega)\right|^2 Q_{A_p}+$$
$$+\left|(\lambda_6-i\omega)\left(-\lambda_2\lambda_9 N_C\frac{\partial A}{\partial M}+\lambda_{10}\left(\lambda_1\frac{\partial A}{\partial M}-i\omega\right)\right)\right|^2 Q_{-B_p}+$$
$$+\left|\frac{\lambda_5}{\lambda_6-i\omega}\left[\lambda_4\left(\lambda_1\frac{\partial A}{\partial M}-i\omega\right)(\lambda_{12}-i\omega)\right]+1\right|^2 Q_{-Y_p}+$$
$$+\left|(\lambda_6-i\omega)(\lambda_{12}-i\omega)(\lambda_1\frac{\partial A}{\partial M}-i\omega)+(\lambda_6-i\omega)(\lambda_2\lambda_9 N_C\frac{\partial A}{\partial M}+\right.$$
$$\left.-\lambda_{10}\left(\lambda_1\frac{\partial A}{\partial M}-i\omega\right))\right|^2 Q_{A,B_p}+$$
$$+\left|\frac{\lambda_5}{\lambda_6-i\omega}\left[\left(\lambda_1\frac{\partial A}{\partial M}-i\omega\right)(\lambda_6-i\omega)(\lambda_{12}-i\omega)+\right.\right.$$
$$\left.\left.\left.-\lambda_4\left(\lambda_1\frac{\partial A}{\partial M}-i\omega\right)(\lambda_{12}-i\omega)\right]-1\right|^2 Q_{A,Y_p}\right)\cdot$$
$$\left|\left(\lambda_1\frac{\partial A}{\partial M}-i\omega\right)\cdot\left[(\lambda_3-i\omega)(\lambda_6-i\omega)(\lambda_{12}-i\omega)-\lambda_{10}\lambda_{11}(\lambda_6-i\omega)+\right.\right.$$
$$\left.\left.-\lambda_4\lambda_5(\lambda_{12}-i\omega)\right]+\lambda_2\lambda_9\lambda_{11}N_C\frac{\partial A}{\partial M}(\lambda_6-i\omega)\right|^{-2} \tag{13}$$
$$S_X(\omega) = \frac{\lambda_7^2 S_{N_{Y_p}}(\omega)+Q_X}{|\lambda_8-i\omega|^2} \tag{14}$$

4 Effect of localisation of CheR on noise power spectrum

Here, we consider the contribution of localisation of enzyme CheR, which is responsible for receptor methylation. We assumed in addition to the model of the full pathway Eq. 1, 2 and 13-17 in the main text, CheR first has to bind to the chemoreceptor complex. We describe localisation of CheR by the following equation

$$\frac{dN_{R,bound}}{dt} = k_{R,on}(N_{R,tot} - N_C N_{R,bound}) - k_{R,off} N_{R,bound} + \eta_{R,bound}, (t) \quad (15)$$

where the first two terms describe binding and unbinding to the receptor complex and the last term fluctuations associated with localisation. $N_{R,tot}$ is the total number of CheR molecules in a cell, N_C the number of receptor complexes, and $N_{R,bound}$ is the number of bound CheR per complex. Hence, $N_{R,tot} - N_C N_{R,bound}$ is the number of unbound CheR molecules free to bind to a complex. The noise term $\eta_{R,bound}$ is assumed to be Gaussian and white, with zero mean and intensity is given by $Q_{R,bound} = 2k_{R,off} N^*_{R,bound}$. Ref. [1] provides an estimate for $k_{R,off} = 0.068$/s for the unbinding of CheR, as well as the steady-state ratio of free and bound CheR of 1:1.86. From this we can calculate $k_{R,on} = k_{R,off} N^*_{R,bound} N_C/(N_{R,tot} - N_C N^*_{R,bound})$, and the characteristic frequency of CheR localisation to be $\lambda_R = k_{R,on} N_C + k_{R,off} = 31.7$/s. The power spectrum of $N_{R,bound}$ is

$$S_{R,bound} = \frac{Q_{R,bound}}{\omega^2 + \lambda_R^2}. \quad (16)$$

The equation of receptor methylation and demethylation Eq. 3 in the main text has to be modified to include bound CheR molecules only affecting receptor methylation:

$$\frac{dM_j}{dt} = \gamma'_R N_{R,bound}(N - A_j) - \frac{\gamma_B}{V_{cell}^2} A_j N_{B_p}^2 + \eta_{M_j}(t). \quad (17)$$

We define another parameter $\lambda^* = \gamma'_R(N - A_j^*)$. We assumed that the methylation rate constant $\gamma'_R N_{R,bound} = \gamma_R$, where γ_R is the methylation rate constant from the full pathway model without considering CheR localisation.

After linearisation, Fourier transformation and rearrangement of equations, we obtain for the contribution to the power spectrum of CheA-P from localisation of CheR

$$\begin{aligned} S_{N_{A_p},R,bound}(\omega) = & \left(\left| (\lambda_6 - i\omega)(\lambda_{12} - i\omega)(-\lambda_2\lambda^*) N_C \frac{\partial A}{\partial M} \right|^2 S_{R,bound}(\omega) \right) \cdot \\ & \left| \left(\lambda_1 \frac{\partial A}{\partial M} - i\omega \right) \cdot [(\lambda_3 - i\omega)(\lambda_6 - i\omega)(\lambda_{12} - i\omega) - \lambda_{10}\lambda_{11}(\lambda_6 - i\omega) + \right. \\ & \left. -\lambda_4\lambda_5(\lambda_{12} - i\omega)] + \lambda_2\lambda_9\lambda_{11} N_C \frac{\partial A}{\partial M}(\lambda_6 - i\omega) \right|^{-2}, \quad (18) \end{aligned}$$

and the contribution to the power spectrum of the motor is

$$\begin{aligned} S_{X,R,bound}(\omega) &= \frac{\lambda_7^2}{|\lambda_8 - i\omega|^2} \cdot \frac{\lambda_5^2(\omega)}{|\lambda_6 - i\omega|^2} \cdot \left(\left| (\lambda_6 - i\omega)(\lambda_{12} - i\omega)(-\lambda_2 \lambda^*) N_C \frac{\partial A}{\partial M} \right|^2 S_{R,bound}(\omega) \right) \cdot \\ & \left| \left(\lambda_1 \frac{\partial A}{\partial M} - i\omega \right) \cdot \left[(\lambda_3 - i\omega)(\lambda_6 - i\omega)(\lambda_{12} - i\omega) - \lambda_{10}\lambda_{11}(\lambda_6 - i\omega) + \right. \right. \\ & \left. \left. -\lambda_4 \lambda_5 (\lambda_{12} - i\omega) \right] + \lambda_2 \lambda_9 \lambda_{11} N_C \frac{\partial A}{\partial M} (\lambda_6 - i\omega) \right|^{-2}. \end{aligned} \tag{19}$$

Figure 9A in the main text shows the effect of CheR localisation on noise spectra of the motor.

5 Effect of CheR gene expression on noise power spectrum

Here, we consider the contribution of CheR gene expression noise. We use the following equation to describe cheR mRNA production and degradation, as well as fluctuations:

$$\frac{dm}{dt} = k_m - \tau_m^{-1} m + \eta_m(t). \tag{20}$$

The parameters $k_m = 0.02$ / min and $\tau_m = 1.5$ min were taken from Ref. [2]. Following mRNA transcription, CheR protein is produced via translation. We use the following equation to describe CheR protein production and degradation, as well as fluctuations:

$$\frac{dN_R}{dt} = k_p m - \tau_p^{-1} N_R + \eta_p(t). \tag{21}$$

The parameter $\tau_p = 79.3$ min was estimated from Ref. [2] and $k_p = \tau_p N_{R,tot}/m^*$ adjusted to give the CheR concentration of 0.2 μM [3]. The noise terms η_m and η_p are assumed to be Gaussian and white, with zero mean and intensities given by $Q_m = 2k_m$ and $Q_p = 2k_p m$, respectively.

The spectrum of the number of CheR molecules N_R is then

$$S_{R,gene}(\omega) = \frac{k_p^2 Q_m/(\omega^2 + \tau_m^{-2}) + Q_p}{\omega^2 + \tau_p^{-2}}. \tag{22}$$

The equation of receptor methylation and demethylation Eq. 3 in the main text has to be modified to include the number of CheR molecules:

$$\frac{dM_j}{dt} = \gamma_R' N_R/N_C(N - A_j) - \frac{\gamma_B}{V_{cell}^2} A_j N_{B_p}^2 + \eta_{M_j}(t). \tag{23}$$

We assumed that the methylation rate constant $\gamma_R' N_R^*/N_C = \gamma_R$, where γ_R is the methylation rate constant from the full pathway model in the main text.

After linearisation, Fourier transformation and rearrangement of equations, we obtain for the contribution to the power spectrum of CheA-P from CheR gene expression

$$\begin{aligned} S_{N_{A_p},R,gene}(\omega) &= \left(\left|(\lambda_6 - i\omega)(\lambda_{12} - i\omega)(-\lambda_2\lambda^*)N_C\frac{\partial A}{\partial M}\right|^2 S_{R,gene}(\omega)\right) \cdot \\ &\left|\left(\lambda_1\frac{\partial A}{\partial M} - i\omega\right) \cdot \left[(\lambda_3 - i\omega)(\lambda_6 - i\omega)(\lambda_{12} - i\omega) - \lambda_{10}\lambda_{11}(\lambda_6 - i\omega) + \right.\right. \\ &\left.\left. -\lambda_4\lambda_5(\lambda_{12} - i\omega)\right] + \lambda_2\lambda_9\lambda_{11}N_C\frac{\partial A}{\partial M}(\lambda_6 - i\omega)\right|^{-2}, \end{aligned} \tag{24}$$

and the contribution to the power spectrum of the motor is

$$\begin{aligned} S_{X,R,gene}(\omega) &= \frac{\lambda_7^2}{|\lambda_8 - i\omega|^2} \cdot \frac{\lambda_5^2(\omega)}{|\lambda_6 - i\omega|^2} \cdot \left(\left|(\lambda_6 - i\omega)(\lambda_{12} - i\omega)(-\lambda_2\lambda^*)N_C\frac{\partial A}{\partial M}\right|^2 S_{R,gene}(\omega)\right) \cdot \\ &\left|\left(\lambda_1\frac{\partial A}{\partial M} - i\omega\right) \cdot \left[(\lambda_3 - i\omega)(\lambda_6 - i\omega)(\lambda_{12} - i\omega) - \lambda_{10}\lambda_{11}(\lambda_6 - i\omega) + \right.\right. \\ &\left.\left. -\lambda_4\lambda_5(\lambda_{12} - i\omega)\right] + \lambda_2\lambda_9\lambda_{11}N_C\frac{\partial A}{\partial M}(\lambda_6 - i\omega)\right|^{-2}. \end{aligned} \tag{25}$$

Figure 9A in the main text shows the effect of gene expression on noise spectra of the motor.

6 Effect of correlations in ligand concentration on noise power spectrum

In the main text, we assumed that fluctuations in ligand concentration only affect individual receptor complexes and that there are no correlations of ligand-induced activity fluctuations between different receptor complexes. Here, we consider the other extreme that each receptor complex experiences the same ligand fluctuations and that there are absolute correlations of fluctuations between receptor complexes. Hence, we assume that the whole receptor cluster is the detection device, i.e. the spectrum of fluctuations of the number of ligand molecules and the concentration, respectively, is (cf. Eq. 33 and 34 in the main text)

$$S_L(\omega) = \frac{Dsc_0}{\omega^2 + k_D^2}, \tag{26}$$

$$S_c(\omega) = \frac{S_L(\omega)}{s^6} \tag{27}$$

with $s = 5 \cdot 10^{-7}$ m the dimension of the whole receptor cluster, i.e. scaled as $\sqrt{N_C}$ compared to the dimension of an individual receptor complex. All other parameters are kept the same. To calculate the total noise at the level of receptor activity in a cell, we now sum up coherently all individual noises at receptor complexes resulting in

$$S_{A_c,ligand}(\omega) = N_C^2\frac{\omega^2\left(\frac{\partial A}{\partial c}\right)^2 S_c(\omega)}{\omega_M^2 + \omega^2}. \tag{28}$$

Figure 9B in the main text shows the comparison of the noise spectra of correlated and uncorrelated ligand noise as transmitted to the motor.

7 Number of high-frequency filters

In Fig. 3A in the main text it is apparent that our model does not fully reproduce the high-frequency response. The high-frequency response seems to be a third-order filter in the frequency range shown, while our model only produces a second-order filter due to CheY-P and motor-switching dynamics. A third filter due to the autophosphorylation dynamics, which is included in our model, becomes only relevant at higher frequencies. Here, we discuss where an additional filter could originate.

We explicitly consider the CheY-P/CheZ binding step, and write down the equations for the dynamics of the concentration of CheY-P, denoted by y, and of CheY-P/CheZ complex, ζ,

$$\frac{dy}{dt} = g_Y\,(Y_{tot}-y)\,a - g_1\,(Z_{tot}-\zeta)\,y + g_2\zeta \tag{29}$$

$$\frac{d\zeta}{dt} = g_1(Z_{tot}-\zeta)y - (g_2+g_3)\,\zeta, \tag{30}$$

where a is the concentration of phosphorylated CheA and g_i are the rates of phosphorylation of CheY (g_Y), CheY-P/CheZ complex formation (g_1), dissociation of CheY-P/CheZ complexes (g_2) and CheY-P dephosphorylation (g_3). Linearising around the steady state (a^*, y^*, ζ^*) yields

$$\frac{d(\Delta y)}{dt} = \underbrace{g_Y\,(Y_{tot}-y^*)}_{\tilde\lambda_1}\Delta a - \underbrace{[g_Y a^* + g_1\,(Z_{tot}-\zeta^*)]}_{\tilde\lambda_2}\Delta y + \underbrace{(g_1 y^* + g_2)}_{\tilde\lambda_3}\Delta\zeta \tag{31}$$

$$\frac{d(\Delta\zeta)}{dt} = -\underbrace{[g_1 y^* + g_2 + g_3]}_{\tilde\lambda_3+g_3}\Delta\zeta + \underbrace{g_1\,(Z_{tot}-\zeta^*)}_{\tilde\lambda_4}\Delta y. \tag{32}$$

Hence, we obtain for the Fourier transform of deviations in the CheY-P concentration

$$\Delta\hat{y} = \frac{\tilde\lambda_1(-i\omega+\tilde\lambda_3+g_3)}{(-i\omega+\tilde\lambda_2)(-i\omega+\tilde\lambda_3+g_3)-\tilde\lambda_3\tilde\lambda_4}\Delta\hat{a}. \tag{33}$$

To make the analysis easier, we can factorise the polynomial in the denominator,

$$\Delta\hat{y} = \frac{\tilde\lambda_1(-i\omega+\tilde\lambda_3+g_3)}{(-i\omega+a_1)(-i\omega+a_2)}\Delta\hat{a}, \tag{34}$$

with $a_{1,2} = (\tilde\lambda_3+g_3+\tilde\lambda_2)/2 \pm \sqrt{(\tilde\lambda_3+g_3+\tilde\lambda_2)^2/4 - \tilde\lambda_2(\tilde\lambda_3+g_3)+\tilde\lambda_3\tilde\lambda_4}$.

We are interested in the behaviour of the frequency-dependent prefactor. Specifically, we ask if by considering the CheY-P/CheZ complex formation we obtain an additional high-frequency filter compared to Eq. 8. It is obvious from Eq. 34 that under most parameter combinations we obtain $1/\omega$ behaviour at

high frequencies. Hence, no additional filter is introduced. A special case appears for $\tilde{\lambda}_3 + k3 \gg a_1,\, a_2$. In this case, a $1/\omega^2$ behaviour is observed for medium frequencies $\max(a_1,\, a_2) \gg \omega \gg \tilde{\lambda}_3 + g_3$. Hence, additional filter appears. At high frequencies $\omega > \tilde{\lambda}_3 + g_3$, the prefactor has $1/\omega$ behaviour. However, analysing the expressions for a_1 and a_2 reveals that $\max(a_1,\, a_2)$ is always greater or equal to $(\tilde{\lambda}_3 + g_3 + \tilde{\lambda}_2)/2$. Therefore, this case does not occur for our dynamics. In conclusion, considering CheY-P/CheZ complex formation does not introduce an additional high-frequency filter.

Other processes in the signalling pathway neglected here are the oligomerisation of CheY-P/CheZ complexes [4–6] and a potential slow release of CheY-P from the sensory complex as discussed in Ref. [7]. Oligomerisation of CheY-P/CheZ complexes for efficient dephosphorylation is similar to CheY-P/CheZ complex formation considered above, and by a similar discussion does not introduce an additional high-frequency filter. However, a delayed release of CheY-P represents effectively a step between CheY phosphorylation and motor switching in the signalling pathway, and hence could introduce a relevant filter if the process is sufficiently slow. Another possibility to explain the steep frequency-dependence of the response function is that the duration of experimental pulses was long enough to leave a signature.

8 Alternative Master-equation approach

Alternative to the Langevin approach, which assumes small fluctuations, we can write down a Master equation for the pathway. Here, we focus on ligand and methylation dynamics at the receptor cluster. Each state of the pathway is described by the variables $[L_j,\, M_j]$ at each of the receptor complexes, where $L_j = c_j s^3$ is the number of molecules in a small volume at the receptor complex and M_j the total methylation level of the receptor complex. Assuming for simplicity only one receptor complex, the Master equation for the probability density p is

$$\begin{aligned}\frac{\partial p(L,M,t)}{\partial t} &= k_D(s^3 c_0)p(L-1,M,t)\\ &+k_D(L+1)p(L+1,M,t)\\ &+\gamma_R[1-A(L,M-1)]p(L,M-1,t)\\ &+\gamma_B[A(L,M+1)]^3 p(L,M+1,t)\\ &-\{k_D(s^3c_0+L)+k_D(s^3c_0)+\gamma_R[1-A(L,M)]+\gamma_B[A(L,M)]^3\}p(L,M,t) \qquad (35)\end{aligned}$$

For small noise, using van Kampen's Ω expansion [8] for the variances of fluctuations in the number of ligand molecules and the methylation level at steady state [9], we obtain

$$\langle \delta L^2 \rangle = c_0 s^3 \tag{36}$$

$$\langle \delta M^2 \rangle = \frac{1}{(3-2A^*)\beta} + \frac{\gamma_R (3-2A^*)\left(\frac{\partial A}{\partial c}\right)^2 c}{A^{*2}(1-A^*)[k_D + \gamma_R(3-2A^*)(1-A^*)\beta]\beta}, \tag{37}$$

with $\beta = 1/2$ the free-energy difference due to adding one methyl group (in units of $k_B T$). The first term is due to fluctuations in the rate of methylation and demethylation and the second term is due to transmitted fluctuations in the activity from ligand noise. This corresponds to the results from the Langevin approach. Specifically, for ligand fluctuation we obtain the same variance after integration of the power spectrum Eq. 26 in the main text. Furthermore, the power spectrum of the receptor complex methylation level using the simplified model from the main text is

$$S_M(\omega) = \frac{Q_M + (\gamma_R + 3\gamma_B A^{*2})^2 \left(\frac{\partial A}{\partial c}\right)^2 S_c(\omega)}{\omega^2 + \omega_M^2}. \tag{38}$$

The variance of the methylation level, obtained by integration of the power spectrum, corresponds to the above result.

9 Langevin model for motor dynamics

We chose to describe the dynamics of the motor using the Langevin Eq. 4 in the main text

$$\frac{dX}{dt} = k_+(1-X) - k_- X + \eta_X(t) \tag{39}$$

with switching rates from CCW to CW (first term) and from CW to CCW (second term), as well as an additive Gaussian white noise term (last term) with zero mean and autocorrelation $\langle \eta_X(t)\eta_X(t')\rangle = Q_X \delta(t-t')$ with $Q_X = 2k_+(1-P_t^*) = 2k_+k_-/(k_+ + k_-)$. For constant switching rate constants k_+ and k_-, the power spectrum X is (cf. Eq. 9 in the main text)

$$S_X(\omega) = \frac{Q_X}{\omega^2 + (k_+ + k_-)^2}. \tag{40}$$

To see that this is a valid description of the binary motor-switching process, we calculate the spectrum exactly according to the derivation in Ref. [10]. For a stochastic two-state process, whose time interval lengths in each of the two states τ_1 and τ_2, respectively, are independent and identically distributed random variables, the power spectrum is given in terms of the Fourier transforms of the waiting time distributions $\Theta_1(\omega)$ and $\Theta_2(\omega)$ for each of the states,

$$S(\omega) = \frac{2}{\omega^2(\langle \tau_1 \rangle + \langle \tau_2 \rangle)} \Re \frac{[1-\Theta_1(\omega)][1-\Theta_2(\omega)]}{1-\Theta_1(\omega)\Theta_2(\omega)}, \tag{41}$$

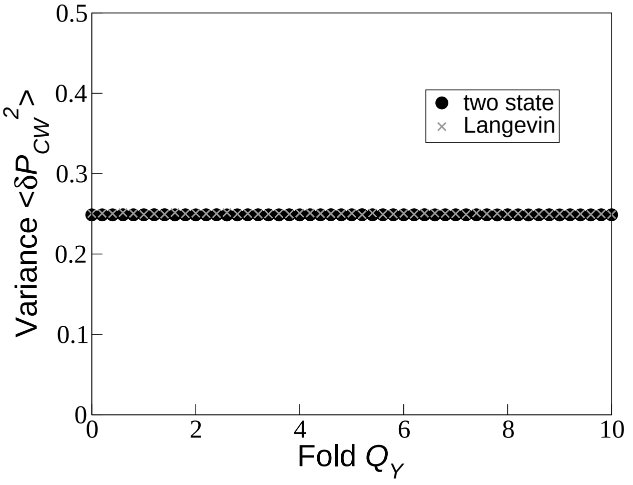

Figure 1: Variance of the motor bias as a function of CheY-P noise intensity $\alpha Q_Y = 2\alpha k_Y$ for Langevin and two-state dynamics.

where $\Re$ indicates the real part. Assuming for the motor that switching between the states CW and CCW, respectively, follows exponential interval distributions determined by rates k_+ and k_- [11, 12], the Fourier transforms of the waiting time distributions are given by,

$$\Theta_{CW}(\omega) = \frac{k_+}{k_+ - i\omega}, \tag{42}$$

$$\Theta_{CCW}(\omega) = \frac{k_-}{k_- - i\omega}, \tag{43}$$

and the power spectrum is

$$S_2(\omega) = \frac{2k_+k_-}{(k_+ + k_-)} \frac{1}{\omega^2 + (k_+ + k_-)^2}. \tag{44}$$

This result is equivalent to the spectrum obtained from the Langevin equation. Furthermore, we tested numerically that the statistics of the Langevin equation and binary process are equivalent for fluctuating rate constants k_+ and k_- due to the CheY-P dynamics. We simulated time courses of CheY-P according to the simplified equation

$$\frac{dN_{Y_p}}{dt} = k_Y - k_{-Y} N_{Y_p} + \eta_{Y_p}(t) \tag{45}$$

with rates $k_Y = 5/s$ and k_Y such that $\langle N_{Y_p} \rangle^* / V_{cell} = \langle Y_p \rangle^* = 3.2\mu M$. The noise term $\eta_{Y_p}(t)$ is Gaussian and white with zero mean and autocorrelation $\langle \eta_{Y_p}(t)\eta_{Y_p}(t') \rangle = 2k_Y \alpha\delta(t - t') \equiv Q_Y \alpha\delta(t - t')$, where we varied α. Fluctuating CheY-P was translated into the rates $k_+(Y_p)$ and $k_-(Y_p)$ according to Fig. 3C in the main text. The Langevin equation was solved using a Euler-Maruyama algorithm [13] and the binary process using a Gillespie algorithm. Figure 1 shows the variances of both processes as obtained from 10^2

runs for each value of α. As can be seen from the figure, the Langevin equation is a good description for the binary process of motor switching.

References

1. Schulmeister S, Ruttorf M, Thiem S, Kentner D, Lebiedz D, Sourjik V: **Protein exchange dynamics at chemoreceptor clusters in *Escherichia coli.*** *Proc Natl Acad Sci U S A* 2008, **105**:6403–6408.
2. Yu J, Xiao J, Ren X, Lao K, Xie XS: **Probing Gene Expression in Live Cells, One Protein Molecule at a Time**. *Science* 2006, **311**:1600–1603.
3. Li M, Hazelbauer GL: **Cellular Stoichiometry of the Components of the Chemotaxis Signaling Complex.** *J Bacteriol* 2004, **186**:3687–3694.
4. Eisenbach M: **Bacterial chemotaxis**. In *Chemotaxis.* Edited by Eisenbach M, Imperial College Press 2004:53–215.
5. Blat Y, Eisenbach M: **Oligomerization of the Phosphatase CheZ Upon Interaction with the Phosphorylated Form of CheY**. *J Biol Chem* 1996, **271**:1226–1231.
6. Blat Y, Eisenbach M: **Mutants with Defective Phosphatase Activity Show No Phosphorylation-dependent Oligomerization of CheZ**. *J Biol Chem* 1996, **271**:1232–1236.
7. Blat Y, Gillespie B, Bren A, Dahlquist FW, Eisenbach M: **Regulation of phosphatase activity in bacterial chemotaxis**. *J Mol Biol* 1998, **284**:1191–1199.
8. van Kampen NG: *Stochastic processes in physics and chemistry.* North Holland 2007.
9. Aquino G, Clausznitzer D, Tollis S, Endres RG: **Optimal receptor-cluster size determined by intrinsic and extrinsic noise.** *Phys Rev E* 2011, **83**:021914.
10. Stratonovich RL: *Topics in the Theory of Random Noise*, New York: Gordon and Breach, *Volume I* 1963 chap. 6, :143–176.
11. Block SM, Segall JE, Berg HC: **Adaptation kinetics in bacterial chemotaxis.** *J Bacteriol* 1983, **154**:312–323.
12. Bai F, Branch RW, Nicolau DV, Pilizota T, Steel BC, Maini PK, Berry RM: **Conformational Spread as a Mechanism for Cooperativity in the Bacterial Flagellar Switch**. *Science* 2010, **327**:685–689.
13. Kloeden PE, Platen E: *Numerical solution of stochastic differential equations.* Springer-Verlag, Berlin, New York 1992.
